# *Cdkn1a* transcript variant 2 is a marker of aging and cellular senescence

**DOI:** 10.18632/aging.203110

**Published:** 2021-05-25

**Authors:** José Alberto López-Domínguez, Sandra Rodríguez-López, Ulises Ahumada-Castro, Pierre-Yves Desprez, Maria Konovalenko, Remi-Martin Laberge, César Cárdenas, José Manuel Villalba, Judith Campisi

**Affiliations:** 1 Buck Institute for Research on Aging, Novato, CA 94945, USA; 2 Departamento de Biología Celular, Fisiología e Inmunología, Universidad de Córdoba, Campus de Excelencia Internacional Agroalimentario, 14071, Córdoba, Spain; 3 Center for Integrative Biology, Faculty of Sciences, Universidad Mayor, Santiago 2422, Chile; 4 Geroscience Center for Brain Health and Metabolism, Santiago, Chile; 5 Unity Biotechnology Inc., South San Francisco, CA 94080, USA; 6 Department of Chemistry and Biochemistry, University of California, Santa Barbara, CA 93106, USA; 7 Lawrence Berkeley National Laboratory, University of California, Berkeley, CA 94720, USA

**Keywords:** p21, p53, mouse dermal fibroblast, ionizing radiation, doxorubicin

## Abstract

Cellular senescence is a cell fate response characterized by a permanent cell cycle arrest driven primarily the by cell cycle inhibitor and tumor suppressor proteins p16^Ink4a^ and p21^Cip1/Waf1^. In mice, the p21^Cip1/Waf1^ encoding locus, *Cdkn1a*, is known to generate two transcripts that produce identical proteins, but one of these transcript variants is poorly characterized. We show that the *Cdkn1a* transcript variant 2, but not the better-studied variant 1, is selectively elevated during natural aging across multiple mouse tissues. Importantly, mouse cells induced to senescence in culture by genotoxic stress (ionizing radiation or doxorubicin) upregulated both transcripts, but with different temporal dynamics: variant 1 responded nearly immediately to genotoxic stress, whereas variant 2 increased much more slowly as cells acquired senescent characteristics. Upon treating mice systemically with doxorubicin, which induces widespread cellular senescence *in vivo*, variant 2 increased to a larger extent than variant 1. Variant 2 levels were also more sensitive to the senolytic drug ABT-263 in naturally aged mice. Thus, variant 2 is a novel and more sensitive marker than variant 1 or total p21^Cip1/Waf1^ protein for assessing the senescent cell burden and clearance in mice.

## INTRODUCTION

The stringent cell growth arrest associated with cellular senescence is determined, among other mechanisms, by activities of cyclin-dependent kinase inhibitor proteins p16^Ink4a^ and p21^Cip1/Waf1^, encoded by the *Cdkn2a* and *Cdkn1a* loci, respectively [1]. The increased expression of these proteins is a major hallmark of senescence in most cells, and therefore have become markers of senescence both in culture and *in vivo*. Consistent with the fact that senescent cells increase with age in many mouse and human tissues, *Cdkn2a* (p16^Ink4a^) mRNA levels also increase with age in these tissues [2]. Based on this association, transgenic mice have been generated to detect [3] and selectively eliminate senescent cells *in vivo* [4, 5]. By contrast, despite having a key role in the senescence growth arrest, *Cdkn1a*/p21^Cip1/Waf1^ upregulation during aging is often moderate or absent *in vivo*, and tissue-dependent [2, 6–8]. For example, p21^Cip1/Waf1^ reporter mouse have shown increased reporter activity only in kidneys of 23.5 month-old mice [9]. Further, in most tissues, calorie restriction does not prevent the age-related increase in *Cdkn1a* expression, in contrast to *Cdkn2a* expression [7]. Consequently, the role of *Cdkn1a*/p21^Cip1/Waf1^ as an *in vivo* marker of aging or cellular senescence remains uncertain.

**Figure 1:**
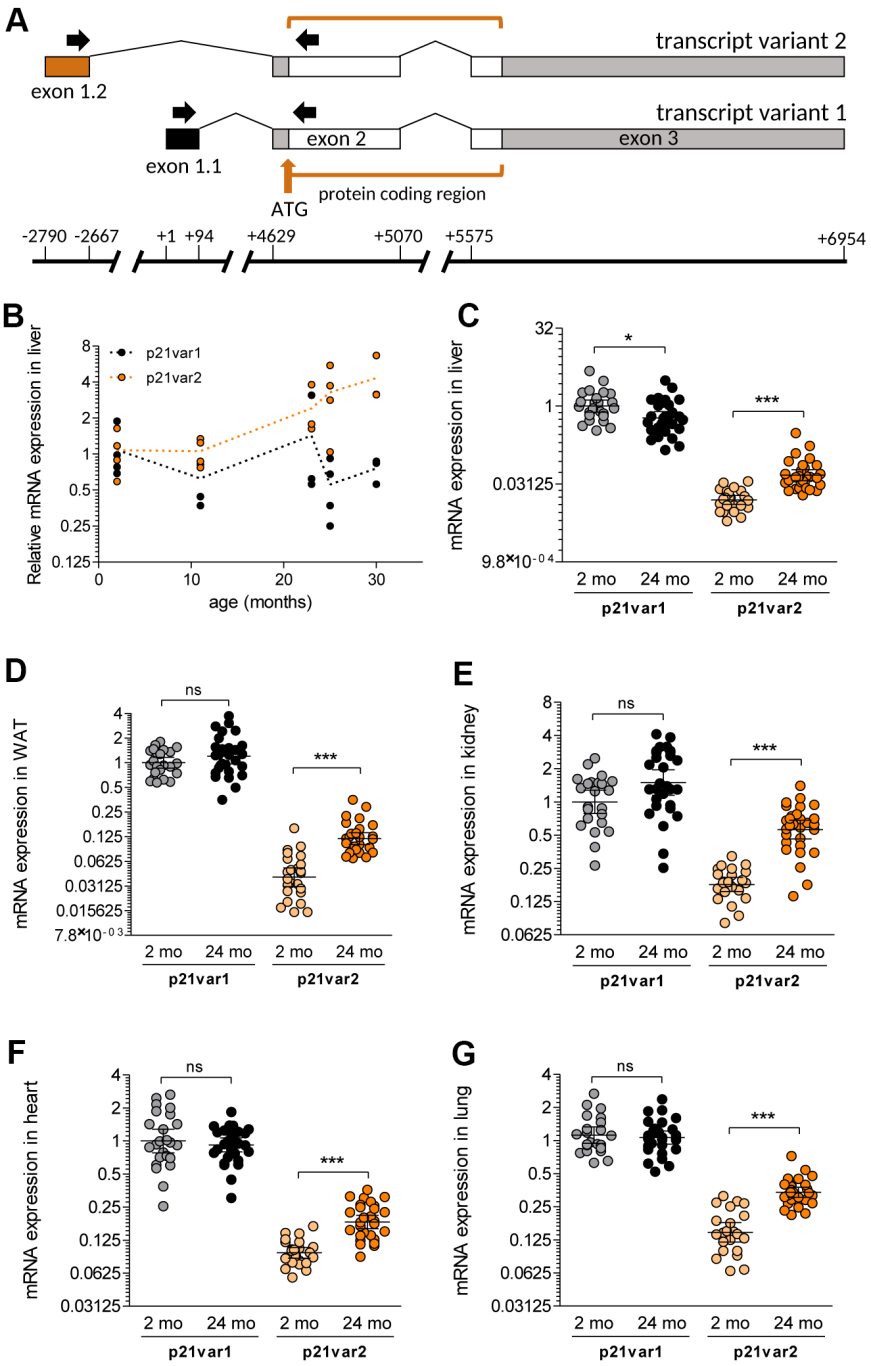
**The *Cdkn1a* variant 2 transcript is preferentially induced during aging.** (**A**) We designed primers (black arrows) to specifically detect *Cdkn1a* variant 1 and 2 transcripts, spanning the first and second exons in each case. The protein-coding region is indicated as well as the ATG start codon (brown arrow). Transcription starts at +1 for p21var1 and at -2790 for p21var2. The first and last bases of each exon are also indicated. (**B**) mRNA levels of p21var1 and p21var2 in the livers of male mice aged 2 to 30 months of age, normalized to levels in livers of 2 month-old animals. (**C**–**G**) Levels of each *Cdkn1a* transcript were assessed in 2 (young) and 24 (old) month-old mice. Animals were young males (n = 12), young females (n = 12), old males (n =14-15) and old females (n = 14-15). Results are shown for (**C**) liver, (**D**) adipose tissue, (**E**) kidney, (**F**) heart and (**G**) lung. In (**C**–**G**) data were normalized to p21var1 levels in young mice. Note Y axes are log-2 scales. 1-way ANOVA and Tukey post-tests were applied. * p < 0.05, ** p < 0.01, *** p < 0.001, ns = not-significant.

Two transcript variants are currently annotated for the murine *Cdkn1a* gene (Figure 1A). The better-studied mRNA (*Cdkn1a* transcript variant 1, NM_007669.5, hereafter termed p21var1) contains three exons, the two latter of which encode the p21^Cip1/Waf1^ protein. An alternative transcript (*Cdkn1a* transcript variant 2, NM_001111099.2, hereafter termed p21var2) differs from p21var1 in the first exon and therefore contains an almost entirely different 5’ untranslated region (UTR), despite encoding the same protein [10]. Additionally, the p21var2 transcription start site (TSS) lies ~2.8 kb upstream of p21var1, and thus might be subject to regulation by elements not present in p21var1. Interestingly, the p21var2 is generally less abundant than p21var1, but the translation of the p21var2 transcript increases under nutrient stress; consequently, the relative contribution of each variant to the total pool of p21^Cip1/Waf1^ protein likely varies depending on stress and possibly other conditions [11]. To date, possible changes in the expression of *Cdkn1a* transcript-specific variants during age or cellular senescence have not been explored.

To fill this gap in our knowledge, we explored the expression levels of each *Cdkn1a* transcript variant in several tissues from aged mice. We also analyze their expression levels in a cell culture model of mouse cells subjected to genotoxic stress-induced senescence to evaluate their relative utility as senescence markers both in culture and *in vivo*. Finally, we show that well-established regulators of *Cdkn1a* expression have variant-specific effects, which add a novel level of complexity to the biological roles of p21^Cip1/Waf1^.

## RESULTS

### *Cdkn1a* transcript variant 2 is preferentially induced with age

To assess expression levels of the individual *Ckdn1a* mRNA transcript variants, we designed two primer sets in which the forward primers hybridize with the variant-specific first exons (Figure 1A). To determine whether differential regulation occurs during aging *in vivo*, we analyzed liver samples from male mice at 2, 11, 23, 25 and 30 months of age. Relative to 2 month-old mice, p21var2, but not p21var1, increased after 20 months of age (Figure 1B). We then obtained additional tissues from 2 and 24 month-old male and female animals (n = 12-15 per sex and age). p21var2 levels were higher than p21var1 levels in aged liver, white adipose tissue, kidney, heart and lung (Figure 1C–1G). Steady-state levels of p21var1 remained unaltered with age, and were even slightly reduced with age in liver. On average, p21var2 abundance increased 3-fold with age in liver, kidney and adipose tissue, and 2-fold in heart and lung. Mice at 4 months of age used as young control yielded equivalent results (data not shown). The transcript encoding p16^Ink4a^ also increased with age in all these tissues (Supplementary Figure 1A–1E).

When males and females were analyzed separately, p21var2 increased with age in all tissues and in both sexes (Supplementary Figure 2A–2J), with the only exception of heart in male mice, where the upwards trend did not reach statistical significance (Supplementary Figure 2D). Of note, we detected higher p21var1 levels in the kidney of aged females (Supplementary Figure 2H). Despite the propensity of p21var2 to increase with age, overall, in all organs tested, p21var1 was more abundant than p21var2, as reported [10]. In sum, p21var2 expression is consistently elevated with age, in contrast with an absence of age-related change in p21var1 levels.

### Both *Cdnk1a* transcript variants are induced in cellular senescence

To determine whether either transcript variant is preferentially upregulated in senescent cells, we induced senescence in primary mouse dermal fibroblasts (MDFs) using 15 GY ionizing radiation (IR), which induces a senescence response in virtually all the irradiated cells. Seven days later, irradiated, but not sham-irradiated, MDFs showed hallmarks of senescence, including increased levels of the mRNA encoding p16^Ink4a^ and lower levels of the mRNA encoding lamin-B1, as expected [12] (Supplementary Figure 3A–3B). The levels of both p21var1 and p21var2 also increased. However, as reported [10], p21var2 levels were 6- to 8-fold lower compared to p21var1 (data not shown). These data show that both of the murine *Ckdn1a* transcript variants are valid readouts to evaluate cellular senescence in cultured MDFs.

**Figure 2:**
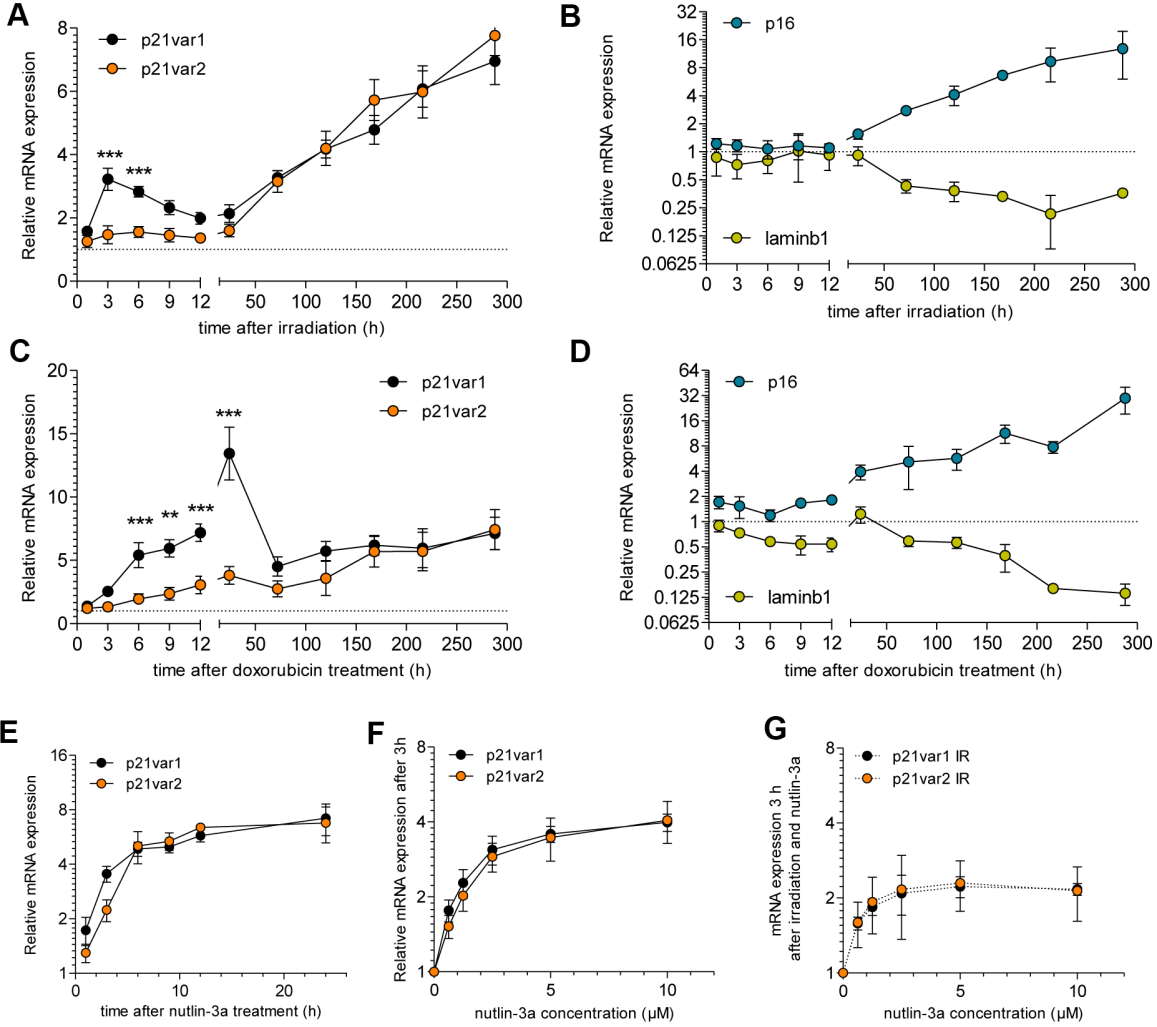
***Cdkn1a* variant 2 increases as cells acquire senescent phenotypes in culture.** Time course of (**A**) p21var1 and p21var2 levels and (**B**) p16^Ink4a^ and lamin B1 mRNA levels after 15 Gy irradiation. 2-way ANOVA test was applied. Time course of (**C**) p21var1 and p21var2 levels and (**D**) p16^Ink4a^ and lamin B1 levels after a 24 h exposure to 250 nM doxorubicin. 2-way ANOVA test was applied. (**E**) p21var1 and p21var2 levels in MDFs after treatment with 10 μM nutlin-3a. (**F**) mRNA levels 3 hours after treatment with increasing doses of nutlin-3a. (**G**) mRNA levels 3 hours after irradiation (15 Gy) and treatment with increasing doses of nutlin-3a. Mean ± SEM is shown. Note Y axes are log-2 scales. * p < 0.05, ** p < 0.01, *** p < 0.001.

We then tested the dynamics of *Cdkn1a* variant expression in MDFs after irradiation. Expression of p21var1 increased 3 hours after irradiation, then progressively declined to a level twice that of baseline by 12 hours after irradiation. In contrast, p21var2 levels remained unaltered for the first 24 hours after irradiation (Figure 2A). Thereafter, both *Cdkn1a* variants steadily increased from day 3, without reaching a plateau by the end of the 12-day time course. Establishment of senescence was verified by increased p16^Ink4a^ and decreased lamin B1 mRNA levels (Figure 2B). Treatment of MDFs with 250 nM doxorubicin (doxo), a chemotherapeutic agent known to cause cellular senescence in culture and *in vivo* [13], increased p21var1 levels within the 24 hours, followed by a smaller increase in p21var2 levels (Figure 2C). Similar to the pattern in irradiated cells, from day 5 onwards both variants were coordinately and increasingly upregulated, concomitant with senescence-associated changes in p16^Ink4a^ and lamin B1 expression (Figure 2D). Together, our results show that p21var1 increases preferentially shortly after acute genotoxic stress, but both variants gradually rise as cells enter a senescent state.

### p53 stabilization upregulates both *Cdkn1a* transcript variants

The basal expression of p21var1 and p21var2 is differentially regulated by p53, likely due to the proximity of the p21var2 transcription start site (TSS) to a p53-response elements (p53-Res) [10]. We performed a promoter analysis (TRANSFAC version 2018.3) for both *Cdkn1a* TSSs, spanning 2.5 kb upstream and 0.5 kb downstream of each TSS (Supplementary Information). Among a plethora of predicted transcription factor binding sites, p53-REs were detected within 500 bases upstream of both TSSs, even when we applied the most stringent algorithm. To understand the p53 responsiveness of the *Cdkn1a* transcript variants, we treated MDFs with 10 μM nutlin-3a, an MDM-2 inhibitor that stabilizes p53 [14]. The levels of both variants increased within 1 hour, reaching a plateau approximately 12 hours later (Figure 2E). Lower concentrations of nutlin-3a failed to reveal any sensitivity differences between the variants. Three hours after treatment, the dose-response curves were similar in shape for both *Cdkn1a* transcripts (Figure 2F). The same was true for senescent (irradiated) MDFs treated with 10 μM nutlin-3a (Figure 2G). These results suggest that p21var1 and p21var2 are equally sensitive to transcriptional upregulation upon p53 stabilization.

### Circadian regulation of p21^Cip1/Waf1^ does not involve transcript variant 2

**Figure 3:**
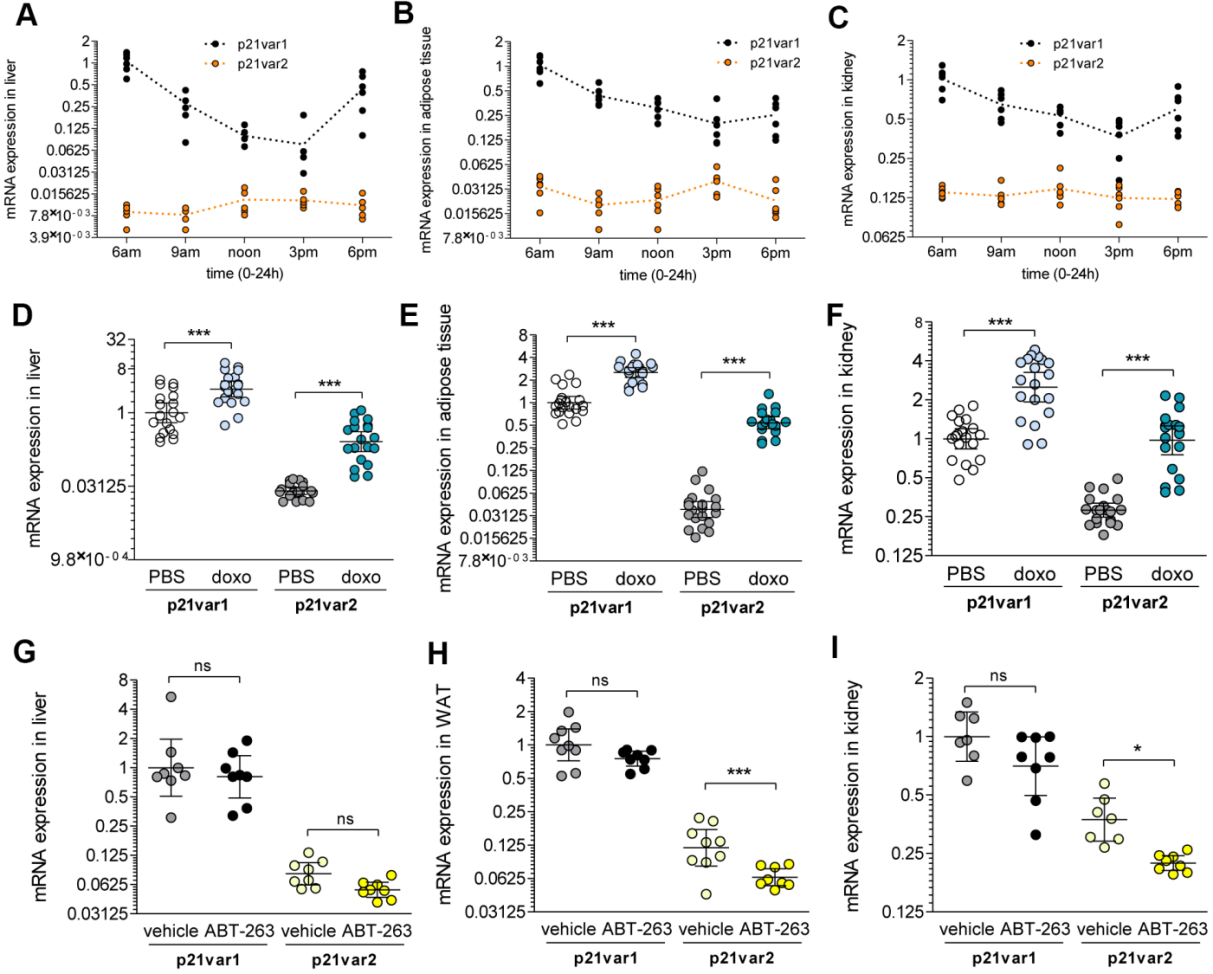
***Cdkn1a* variant 1 is circadian regulated, whereas variant 2 rises with senescence *in vivo*.** (**A**) *Cdkn1a* variants expression throughout the light cycle (6 am to 6 pm) in the liver, (**B**) white adipose tissue and (**C**) kidney of 6 week-old male mice. (**D**–**F**) A cohort of 6 week-old mice were treated with doxorubicin or vehicle (n = 9-10 for either males or females, n = 9-10 for either vehicle or doxorubicin) and *Cdkn1a* variant levels were analyzed 6 weeks later. Results are shown for (**D**) liver, (**E**) adipose tissue and (**F**) kidney. (**G**–**I**) A cohort of 18 to 22 month-old mice were treated with ABT-263 or vehicle. Pooled results are shown for (**G**) liver, (**H**) adipose tissue and (**I**) kidney. 1-way ANOVA and Tukey post-tests were applied. * p < 0.05, ** p < 0.01, *** p < 0.001, ns = not significant.

To further explore the expression pattern of p21var2 *in vivo*, we asked whether it is subject to circadian regulation, as described for p21^Cip1/Waf1^ [15]. We euthanized 2 month-old male mice at 3 hour intervals for 12 hours. In liver samples, p21var1 mRNA levels were highest at the end of the dark cycle (6:00 am Pacific time) and progressively decreased 8-fold to a minimum in the afternoon (Figure 3A). The p21var2 remained unaltered, at lower levels, throughout the same period. A similar pattern was observed in adipose and kidney tissue (Figure 3B, 3C). These results indicate that the circadian regulation of p21^Cip1/Waf1^ is driven solely by expression of *Cdkn1a* transcript variant 1.

### Expression of p21var2 *in vivo* is increased by doxorubicin and decreased by ABT-263

To determine how *Cdkn1a* transcript variants are expressed when cellular senescence is induced *in vivo*, we induced cellular senescence *in vivo* by intraperitoneal injection of a single dose (10 mg/kg) of doxorubicin. After 6 weeks, p21var1 increased 3-fold while p21var2 increased 25-fold (reaching levels comparable to those of p21var1 at baseline) in the livers of treated mice (Figure 3D and Supplementary Figure 4A, 4B). Similar elevations were observed in the adipose tissue and kidneys (Figure 3E, 3F and Supplementary Figure 4C–4F) of male and female mice, consistent with the widespread luminescence reported in doxorubicin-treated p21-reporter mice [9]. Our data suggest that p21var2 is a more sensitive readout for doxorubicin-induced senescence *in vivo* than p21var1.

ABT-263 (navitoclax) has been shown to clear senescent cells by inhibiting Bcl-2 and related anti-apoptotic proteins [16, 17]. In a mixed cohort of 20-22 month-old male and female mice, ABT-263 specifically reduced p21var2 levels in adipose tissue and kidney, whereas p21var1 levels remained unaltered (Figure 3H–3L). There were no significant changes in the levels of either variant in liver (Figure 3G). Together, these results reinforce the idea p21var2 is a better marker than p21var1 for assessing the presence of senescent cells *in vivo*.

## DISCUSSION

*Cdkn1a* transcript variant 2 has received little attention since it was first described, likely because the encoded protein is identical to that encoded by variant 1, and both variants are regulated by p53. Our results are, to our knowledge, the first to study *Ckdn1a* transcript variants in the context of aging. We show that, although tissue-specific exceptions may arise, p21var2 but not p21var1 is a better candidate marker of aging and senescence in mice.

Our findings help explain previous reports regarding changes in p21^Cip1/Waf1^ with age. Thus, given the architecture of the promoters for both transcript variants, a p21^Cip1/Waf1^ reporter mouse that included 2.5 kb of the *Cdkn1a* upstream sequence [9] would not detect p21var2, unlike a reporter mouse containing 4.5 kb of *Cdkn1a* upstream sequences [18] or a knock-in *p21^FLuc^* reporter mouse [19]. In line with our data, the reporter mouse containing 2.5 kb of the *Cdkn1a* promoter did not detect an age-related increase in expression, except for the kidney, in which we also observed an increase in p21var1 levels in old females. Our findings clarify the interpretation of previous and future results on the age-related changes in expression of this locus.

Key to understanding the biological context of *Cdkn1a* transcriptional regulation is the different circadian expression pattern between both transcript variants. Thus, comparisons among previous studies may be confounded by an absence of data regarding the time of euthanasia, a limitation that could be, in principle, avoided by measuring p21var2. In the liver, the wide circadian oscillation of p21var1 contrasts with the more subtle age-related changes in p21var2. This disconnect suggests that p21var1 would be, in accordance to its higher relative contribution to the *Cdkn1a* mRNA pool, the main source of p21^Cip1/Waf1^ for homeostatic functions [9], whereas p21var2 could be more selectively responsive to stress signals and ultimately more reflective of cellular senescence *in vivo*.

Our data do not explain why an increase in p21var2, but not p21var1, which is also observed in cultured senescent cells, is detected in aged animals. One possibility is that some transcriptional modulators are evident *in vivo*, but not necessarily in cultured cells. In addition, there may be cell type-specific expression of *Cdkn1a* variants in senescent cells. Our data show that p53 stabilization cannot explain the expression dynamics of *Cdkn1a* variants upon genotoxic-stress. However, the differences between the variant promoters, of which our analysis provides only an initial hint, may well affect which variant is expressed under different circumstances.

A recent report describes how the dynamics of p21^Cip1/Waf1^ levels in response to DNA damage determine whether cancer cells enter a permanent cell cycle arrest or return to proliferation [20]. It remains unexplored the possibility that the different transcript variants are preferentially associated with one or other cell fate. Additionally, translational regulation at the 5’ UTR of the variants has been shown to affect cell fate [11, 21]. Human cells also express several *Cdkn1a* transcript variants [22]. Among the ten human transcript variants currently annotated, at least one (transcript variant 4) shares translational regulatory mechanisms with the murine p21var2 [21]. Interestingly, even though murine variant 2 and human variant 4 do not appear to share sequence homology, the translational regulation in both transcripts is driven by the integrated stress response and results in cell cycle arrest [21]. The potential relevance of this mechanism for cellular senescence in humans remains unknown, and the functions and interrelations of the different *Cdkn1a* transcript variants have not been studied in depth. Our findings may thus lead to a better understanding of the age-related functions of p21 and improve our ability to monitor the effectiveness of anti-aging therapies.

## MATERIALS AND METHODS

### Mouse models

### Animal husbandry

C56BL/6 mice were purchased from The Jackson Laboratory and allowed to acclimate to the Buck Institute facilities. The animals were group housed under controlled conditions of temperature (22-24° C), humidity (40-60%), and a 12 h light-dark cycle. All animal procedures were approved by the Buck Institute Institutional Animal Care and Use Committee (IACUC).

### Aging cohorts

C56BL/6 were aged at the Buck Institute vivarium and male and female littermates derived from the same colony were used as young controls.

### Doxorubicin treatment

6-week old male and female C57BL/6 littermates were intraperitoneally (IP) injected with a single dose of doxorubicin (10 mg/kg) to induce widespread senescence [13]. After 6 weeks, the animals were euthanized by CO_2_ inhalation followed by cervical dislocation, and tissues were harvested and flash frozen in liquid nitrogen.

### ABT-263 treatment

ABT-263 or vehicle (5% DMSO, 95% corn oil) was delivered to 20-month old C57BL/6 littermates via IP injection at 50 mg/kg for 7 consecutive days per cycle for two cycles, with a 2-week interval between cycles. Animals were sacrificed one week after the last treatment cycle and tissues were collected and flash frozen in liquid nitrogen.

### Cell culture

### Mouse dermal fibroblasts

Primary mouse dermal fibroblasts (MDF) were obtained from postnatal day 2-3 C57BL/6 mice. Skin was excised from the mice and incubated overnight in 0.25% trypsin-EDTA. The dermis was mechanically disengaged and incubated with 10 mg/ml collagenase for 30 min at room temperature. The mixture was filtered and plated. MDF were cultured in 3% oxygen in Dulbecco’s modified Eagle’s medium (DMEM) with 10% fetal bovine serum and 100 U/ml streptomycin and penicillin.

### Irradiation and doxorubicin treatment

MDFs were induced to senesce by X-irradiation (15 Gy), as described [23], or mock-irradiated (control). Alternatively, MDF were treated with 250 nM doxorubicin or vehicle (DMSO, control) for 24 h, then washed and cultured in fresh complete medium. Senescence markers were tested at different times (1, 3, 6, 9, 12 h and 1, 3, 5, 7, 9, 12 days) after treatment.

### Nutlin-3a treatment

MDFs were incubated with 10 μM nutlin-3a or vehicle (DMSO, control) and RNA was collected 1, 3, 6, 9, 12 and 24 h later. MDFs were also treated with different nutlin-3a concentrations (0.6-10 μM), irradiated (15 Gy X-rays) or mock-irradiated and RNA collected 3 h later.

### RNA extraction and RT-qPCR

RNA was extracted using Tri-reagent and isolated with the Direct-Zol RNA miniprep kit (Genesee Scientific). For adipose tissue, the RNeasy Lipid Tissue mini kit (Qiagen) was used. cDNA was synthesized using the High Capacity cDNA RT kit (Life Technologies). Transcripts were analyzed using a Roche LightCycler 480 II in 384-well plates and the UPL probe system. Bioline SensiFast Probe No-ROX was used as a master mix. Primer sequences and respective probes were as follows: Cdkn2a (p16), forward 5’-TCCTCGCAGTTCGAATCTG, reverse 5’-AACTCTTTCGGTCGTACCCC, with a custom designed probe (5'- /56-FAM/AGG TGA TGA /ZEN/TGA TGG GCA ACG TTC AC/3IABkFQ/ -3'); *Cdkn1a* (p21) variant 1, forward 5’-TCCACAGCGATATCCAGACA, reverse 5’-GGACATCACCAGGATTGGAC, with UPL probe 21; *Cdkn1a* (p21) variant 2, forward 5’-TTGCCAGCAGAATAAAAGGTG, reverse 5’-TTTGCTCCTGTGCGGAAC, with UPL probe 9; β-actin: forward 5’- CTAAGGCCAACCGTGAAAAG, reverse 5’- ACCAGAGGCATACAGGGACA, with UPL probe 64; tubulin: forward 5’- CTGGAACCCACGGTCATC, reverse 5’- GTGGCCACGAGCATAGTTATT, UPL probe 88. Results were normalized to β-actin and tubulin. ΔΔCt values prior to logarithmic transformation were used for statistical analyses.

### Promoter analysis

Transcription factor binding sites were analyzed using TRANSFAC version 2018.3 (geneXplain) and sequences between -2500 and +500 for each variant. For each variant, two analyses were performed, either minimizing false positives (minFP) or balancing false positives and false negatives (minSUM), using algorithms provided by the software.

### Statistical analysis

Unless otherwise noted, results are shown as individual data points with geometric means and 95% CIs. Comparisons between multiple groups were analyzed using 1-way ANOVA with Tukey post-tests, or 2-way ANOVA with Bonferroni post-tests if a second variant (usually time) was present. No statistical outliers were removed.

## Supplementary Material

Supplementary Figures
