# Supplementary material for: Cdkn1a transcript variant 2 is a marker of aging and cellular senescence
Source: Aging (Albany NY). 2021 May 25;13(10):13380–92. doi: 10.18632/aging.203110 (PMC8202863; doi:10.18632/aging.203110)
Supplement: Supplementary Figures [file aging-13-203110-s001.pdf]

SUPPLEMENTARY FIGURES

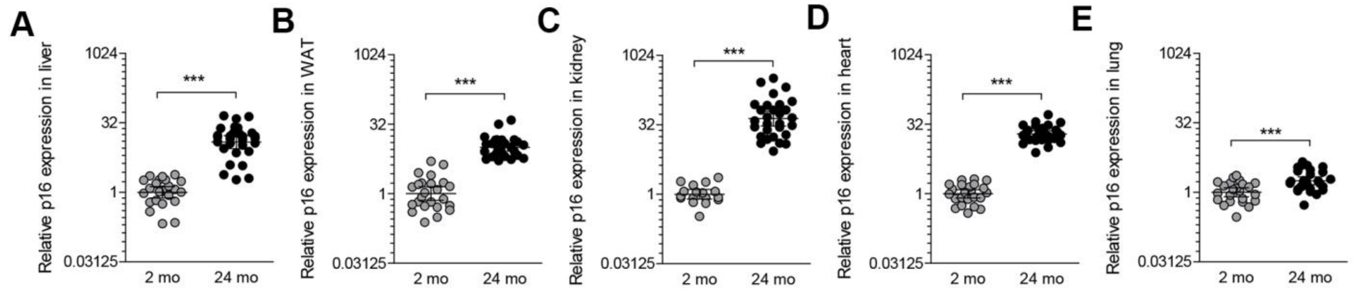

Supplementary Figure 1. p16^{Ink4a} mRNA levels increase with age in multiple tissues. mRNA levels were determined in (**A**) liver, (**B**) white adipose tissue, (**C**) kidney, (**D**) heart and (**E**) lung of 2 and 24 month old mice (n = 24-28). Males and females were pooled. Note Y axes are log-2 scales. t-tests were applied. *** p < 0.001.

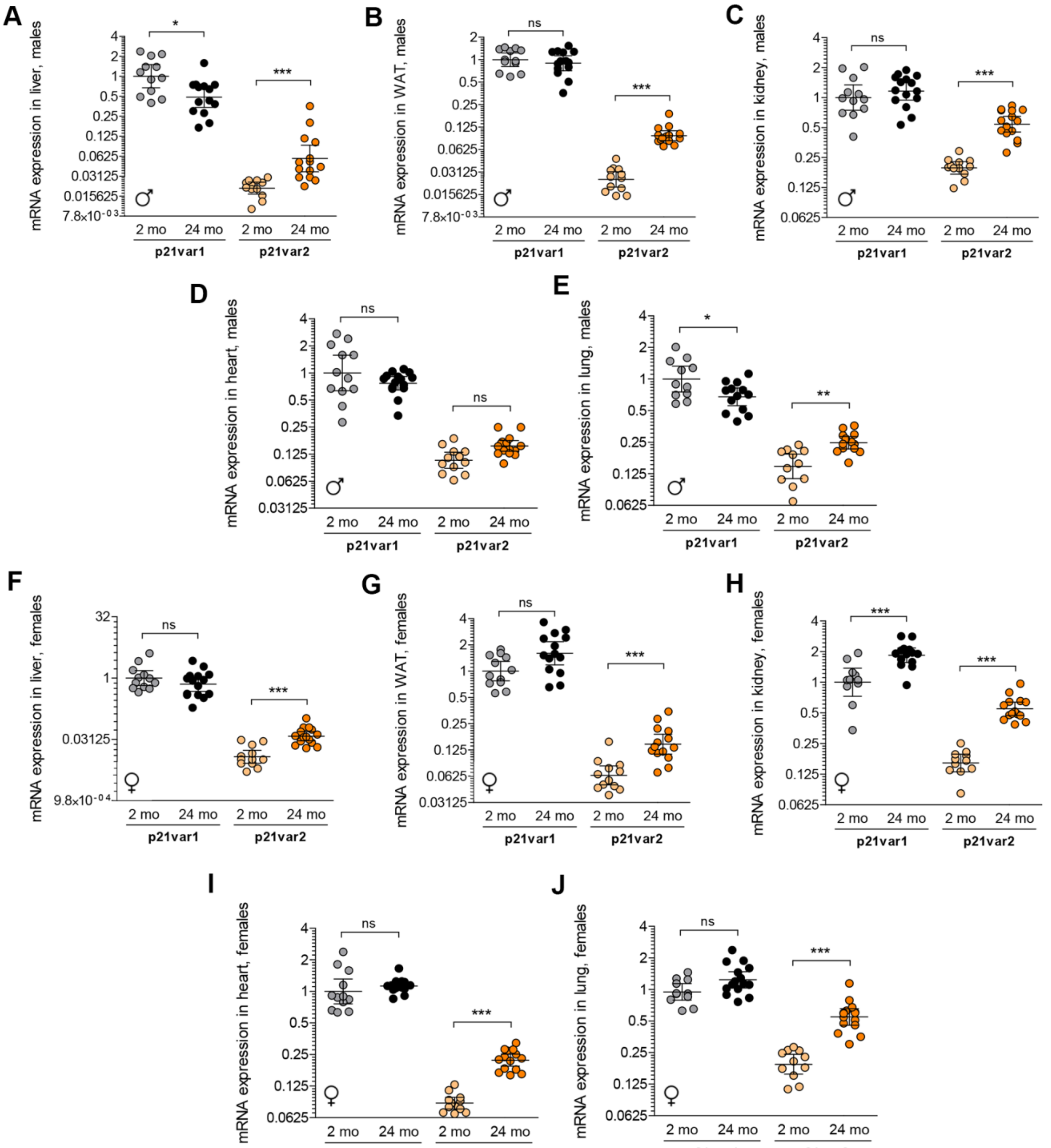

Supplementary Figure 2. *Cdkn1a* transcript variants in young and aged male and female mice. Levels of *Ckdn1a* variants in the (**A**) liver, (**B**) white adipose tissue, (**C**) kidney, (**D**) heart and (**E**) lung of male 2 and 24 month old mice from Figure 1. Expression levels in the (**F**) liver, (**G**) white adipose tissue, (**H**) kidney, (**I**) heart and (**J**) lung of female mice at the same ages. Note Y axes are log-2 scales. 1-way ANOVA and Tukey post-tests were applied. * $p < 0.05$, ** $p < 0.01$, *** $p < 0.001$, ns = not significant.

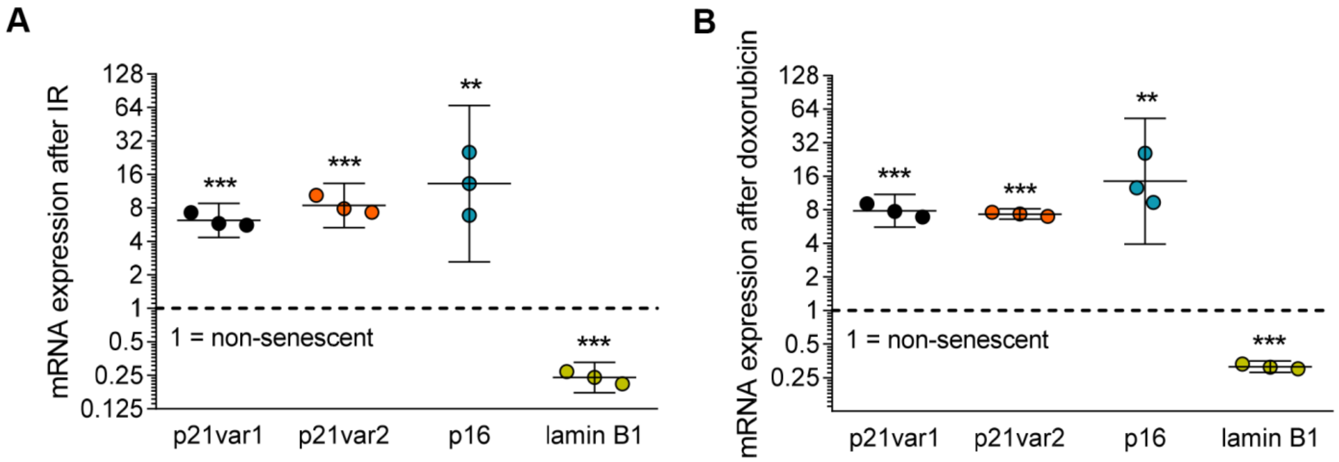

Supplementary Figure 3. Markers of senescence in cultured mouse dermal fibroblasts. (**A**) Levels of *Cdkn1a* transcript variants and p16Ink4a and lamin B1 mRNAs 7 days after irradiation (15 Gy). (**B**) Levels 7 days after a 24 h exposure to 250 nM doxorubicin. Note Y axes are log-2 scales. ** p < 0.01, *** p < 0.001 vs non-senescent control for each transcript (=1).

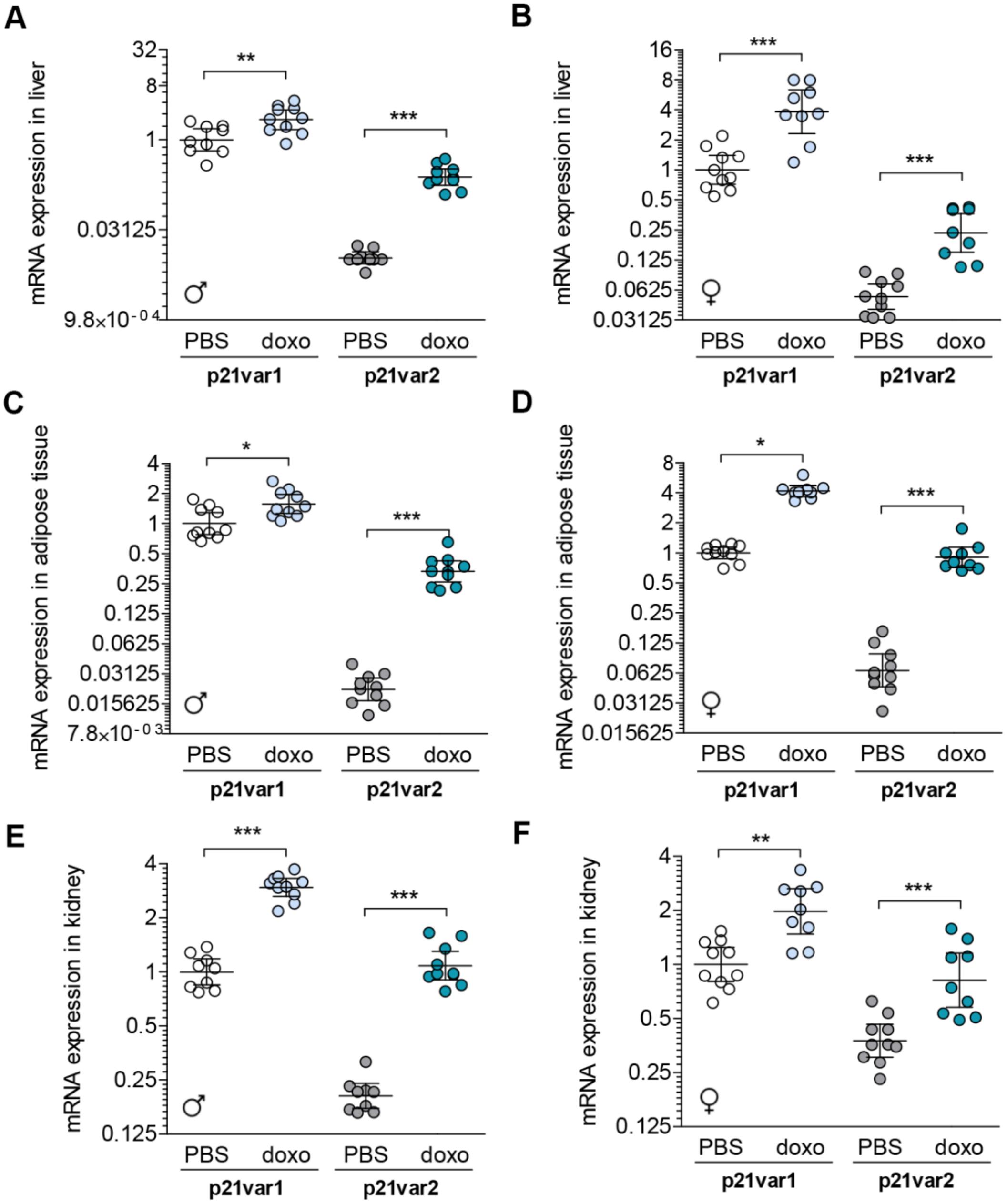

Supplementary Figure 4. *Cdkn1a* transcript variants in doxorubicin-treated male and female mice. Transcript levels were determined in (**A**, **B**) liver, (**C**, **D**) adipose tissue and (**E**, **F**) kidney of male or female 3.5 month old mice, 6 weeks after treatment with doxorubicin (n = 9-10 per sex or treatment). 1-way ANOVA and Tukey post-tests were applied. Note Y axes are log-2 scales. * $p < 0.05$, ** $p < 0.01$, *** $p < 0.001$, ns = not significant.
